# Enhanced growth and recombinant protein production of *Escherichia coli *by a perfluorinated oxygen carrier in miniaturized fed-batch cultures

**DOI:** 10.1186/1475-2859-10-50

**Published:** 2011-06-27

**Authors:** Maciej Pilarek, Julia Glazyrina, Peter Neubauer

**Affiliations:** 1 Department of Biotechnology and Bioprocess Engineering, Faculty of Chemical and Process Engineering, Warsaw University of Technology, Warynskiego 1, 00-645 Warsaw, Poland; 2 Laboratory of Bioprocess Engineering, Department of Biotechnology, Technische Universität Berlin, Ackerstrasse 71-76, D-13355 Berlin, Germany

## Abstract

**Background:**

Liquid perfluorochemicals (PFCs) are interesting oxygen carriers in medicine and biotechnology with a high solubility for oxygen. They have been repeatedly used for improving oxygen transfer into prokaryotic and eukaryotic cell cultures, however their application is still limited. Here we show the great benefit of air/oxygen saturated perfluorodecalin (PFD) for high cell density cultivation of *Escherichia coli *in microwell plates and their positive effect on the soluble production of a correctly folded heterologously expressed alcohol dehydrogenase.

**Results:**

In EnBase^® ^cultivations the best effect was seen with PFD saturated with oxygen enriched air (appr. 10 μM oxygen per ml) when PFD was added at the time of induction. In contrast the effect of PFD was negligible when it was added already at the time of inoculation. Optimisation of addition time and content of loaded oxygen into the PFD resulted in an increased the cell density by 40% compared to control cultures, and correspondingly also the product yield increased, demonstrated at the example of a recombinant alcohol dehydrogenase.

**Conclusions:**

PFCs are a valuable additive in miniaturized cell culture formats. For production of recombinant proteins in low cell density shaken cultures the addition of oxygen-enriched PFD makes the process more robust, i.e. a high product yield is not any more limited to a very narrow cell density window during which the induction has to be done. The positive effect of PFD was even more obvious when it was added during high cell density cultures. The effect of the PFD phase depends on the amount of oxygen which is loaded into the PFD and which thus is a matter of optimisation.

## Background

Perfluorochemicals (PFCs), also known as fluorocarbons or perfluoroalkanes are synthetic fluorine-substituted derivatives of hydrocarbons, i.e. they are similar to hydrocarbons, but all hydrogens are replaced by fluoride. Due to the strength of the carbon-fluoride bond, they are stable and inert compounds with a high resistance to heat [1-3].

Liquid PFCs are characterized by a high solubility of oxygen, carbon dioxide and other non-polar gases which has raised much interest in medical and technical applications [3-5]. Perfluorinated liquids dissolve gases according to Henry's Law and the gas transfer rate into PFCs increases linearly with the partial pressure of a component in the gaseous phase. There is no chemical attraction of oxygen molecules to PFCs in contrast with the sigmoid dissociation curve for biological oxygen carriers (e.g. hemoglobin or myoglobin). Molecules of gases are just occupying cavities between those of the liquid PFCs [2,3,6]. The lack of chemical bonds between oxygen and PFC also allows the easy release of oxygen, e.g. into a contacting water phase. The oxygen solubility in perfluorinated derivatives of hydrocarbons is 35 to 44 mM, which is approximately 20 × higher than the solubility of oxygen in water (2.2 mM). The solubility of carbon dioxide in liquid PFCs can even be up to 3 × higher [2,3].

For medical applications PFC emulsions have been studied as temporary intravascular oxygenation media (so-called "blood substitutes") and as media for preservation of human organs prior to transplantation. Pure liquid PFCs have been evaluated in liquid ventilation studies on premature and new born babies [4,5,7]. In experimental biotechnology various liquid PFCs were applied as carriers of different kinds of gases (O_2_, CO_2_, N_2_O) to supplying them into culture media or as scavengers of gaseous cellular by-products [1-3,6]. Other biotechnological applications of PFCs are related to culturing of 3-D animal cells aggregates on flexible liquid/liquid interfacial created between PFC and culture medium layers [8,9].

The use of PFCs as oxygen carriers in biotechnology has some advantages in comparison with other kinds of oxygen vectors such as chemically modified hemoglobin derivatives, siloxylated copolymers, silicone oils, or hydrocarbons. The lack of toxicity and no negative side-effects of liquid PFCs on various kinds of living cells were confirmed by experimental results and clinical researches [1-3,5]. Liquid PFCs are non-miscible with aqueous media and thus create a separate phase below the aqueous phase which can be effectively recovered from culture systems and then reused. Other merits of perfluorinated oxygen carriers is their heat stability, so that they can be autoclaved and their high chemical stability. Thus they can be stored easily at room temperature.

During the past thirty years many studies have shown that application of a perfluorinated oxygen carrier can facilitate oxygen transport in different types of microbial [1-3,6,10], plant cell [2,10] and animal cell cultures [8,9], however so far this has not resulted in real applications. The reasons are that either the real improvements on growth were minor and especially the relatively high costs of PFCs.

There are only few studies in which PFCs were applied in *Escherichia coli *cultures. About six-fold higher cell densities of *E. coli *were obtained in bioreactor cultures which were periodically aerated by pure perfluoromethylodecalin sprayed into the culture medium and no adverse effects were observed [11]. In another study *E. coli *cells were cultivated in the presence of perfluorotributylamine in emulsified form. The emulsion with 20-50 μm droplets was obtained by intensive mechanical agitation within a bioreactor and the created emulsion was bubble aerated [12]. In this case the cell density increased only by a factor of two.

Biotechnological applications of PFCs are still limited by their relatively high cost, which makes their use in large-scale bioprocesses uneconomically. However, PFCs could be successfully used in miniature-scale cultivations of microbial cells to prevent oxygen limitation during grow to high cell densities, especially in high throughput screening approaches.

High cell density cultures are mostly performed as carbon source limited fed-batch by continuous addition of the carbon source as a highly concentrated liquid at a growth limiting rate. Importantly, to keep the culture in an aerobic state, the feed rate must be balanced with the volumetric oxygen transfer rate, which is limited by the poor solubility of oxygen into the aqueous medium and the technical setup, i.e. the sparging or shaking system. Furthermore, the feed rate has a direct influence on the final cell density. Consequently, approaches that increase the volumetric oxygen transfer rate allow a higher substrate feed rate and can thus result in higher cell densities.

The EnBase^® ^technology is a miniaturized fed-batch cultivation system for high cell density cultivation of microorganisms [13-16]. In this system the controlled supply of glucose is realized by the use of an internally dissolved glucose polymer which cannot be directly assimilated by growing cells and the application of an amylolytic enzyme which releases single glucose monomers. The amount of the enzyme determines the release rate of glucose. By this simple principle EnBase allows the cultivation of cells under glucose-limited fed-batch conditions while the glucose feed rate, and thus the growth rate of the cells, can be simply controlled by the amount of the enzyme. However, the maximum amount of enzyme which can be added depends from the oxygen transfer rate.

Thus our hypothesis for this study was that air/O_2_-saturated liquid PFCs can enhance the cell density and eventually production of recombinant proteins in small-scale high cell density cultivations, and this was tested in 24-deep well plates. The study was performed at the example for *E. coli *for the reason that this organism is widely used and *E. coli *small-scale cultures are widely applied e.g. in the metagenomic area, in high throughput crystallization of proteins, and for creation and screening of gene libraries.

To our knowledge this is the first report of the use of a perfluorinated oxygen carrier in a miniaturized fed-batch system to enhance the yield of cultured cells and protein production and probably also the first report on the application of air/O_2 _saturated PFCs in high cell density bacterial cell cultures in general.

## Results

**Figure 1:**
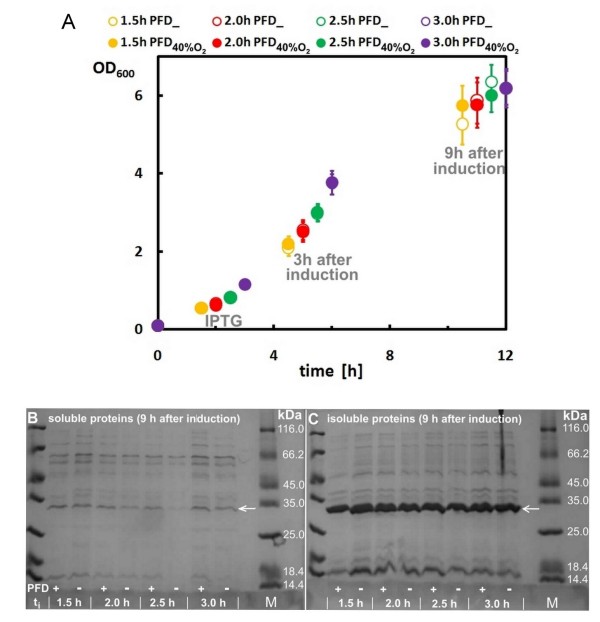
**The comparison of growth (OD_600_)(A) and ADH amounts (B and C) for batch cultures of *E. coli *RB791 *pAdh *on LB-medium supplemented with PFD_40%O2 _and or without PFD (negative control, marked as "PFD_")**. Cultured cells were induced by IPTG at various times (1.5, 2.0, 2.5, and 3.0 h after inoculation). OD_600 _values were measured at the time of induction and after 3 and 9 h after induction. SDS-PAGE analysis of soluble (B) and insoluble (C) protein fractions: t - time of IPTG induction; PFD +/- - cultures supplemented (+) with PFD_40%O2 _or control cultures (-) without PFD; arrows - heterologous alcohol dehydrogenase (ADH).

Firstly, it was our aim to evaluate whether perfluorodecalin (PFD) has an effect on the growth of *E. coli *and production of a heterologous alcohol dehydrogenase (ADH) in complex medium (LB) cultures performed in 24-deepwell plates (DWPs). Therefore *E. coli *RB791 pAdh was grown in DWPs at 30°C. Half of the cultures were cultivated with a bottom phase of PFD which was saturated with oxygen-enriched air (+ 40% v/v O_2_); the other cultures were performed without PFD (as control). All wells of the plate were inoculated at the same time, but different wells were induced at three different time points, representing different cell densities (OD_600 _between 0.4 and 1.2), as we suggested that the effect of PFD might be dependent on the cell density. Although we could not detect any negative effects of PFD on the development of cell density, also no positive effects could be observed as it is shown in Figure 1.

The effect of the different culture conditions on product formation was evaluated by analysing the ADH accumulation in the soluble (correctly folded) or insoluble (inclusion bodies) protein fractions by SDS-PAGE at the end of each experiment (9 hours after induction). Generally, high expression of ADH was observed in all samples. However, whereas the amount of the insoluble protein fraction was almost the same in all samples harvested from 24-DWP cultures on complex medium (LB), clear differences were observed for the amount of soluble ADH, which was especially improved in the PFD containing cultures which were induced at 2.0 or 2.5 h (cf. Figures 1B and 1C).

### PFD in miniaturized fed-batch cultures of *E. coli*

Next experiments were performed as EnBase-Flo miniaturized fed-batch cultures in 24-DWPs. Initial experiments showed that the cell density was not affected when PFD was added from the start of the cultivation, but there was an improved benefit when PFD was added later during the cultivation at higher cell densities (data not shown). Thus we decided to add PFD only after 15 hours of cultivation, to provide the proposed beneficial effect only when the culture's metabolism is activated by addition of the EnBase booster mix and additionally 1.5 GAU L^-1 ^of polymer degrading enzyme, which has been earlier shown to be beneficial for the pH balance and thus resulted in a higher yield of a number of recombinant proteins (see [13]).

**Figure 2:**
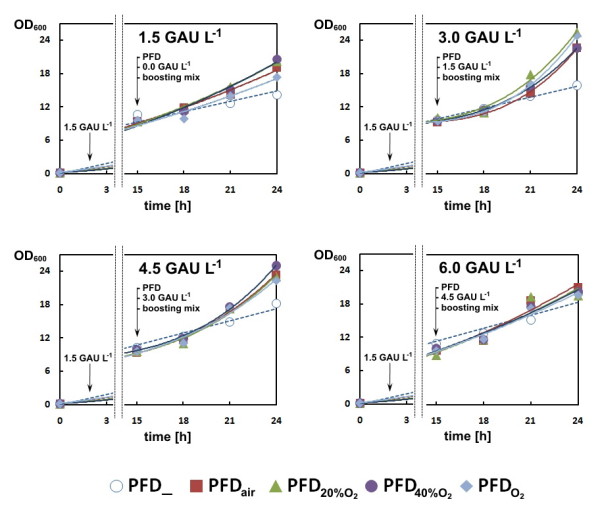
**Growth curves of *E. coli *RB791 *pAdh *cells cultured in EnBase-Flo and supplementation with PFD loaded with different concentrations of O_2 _at the time of induction**. The control culture without PFD is named "PFD_".

In our case, as typical for EnBase cultures all wells reached approximately the same OD_600 _of approximately 10 during the initial 15 hours, i.e. the time of induction and PFD addition (Figure 2).

**Figure 3:**
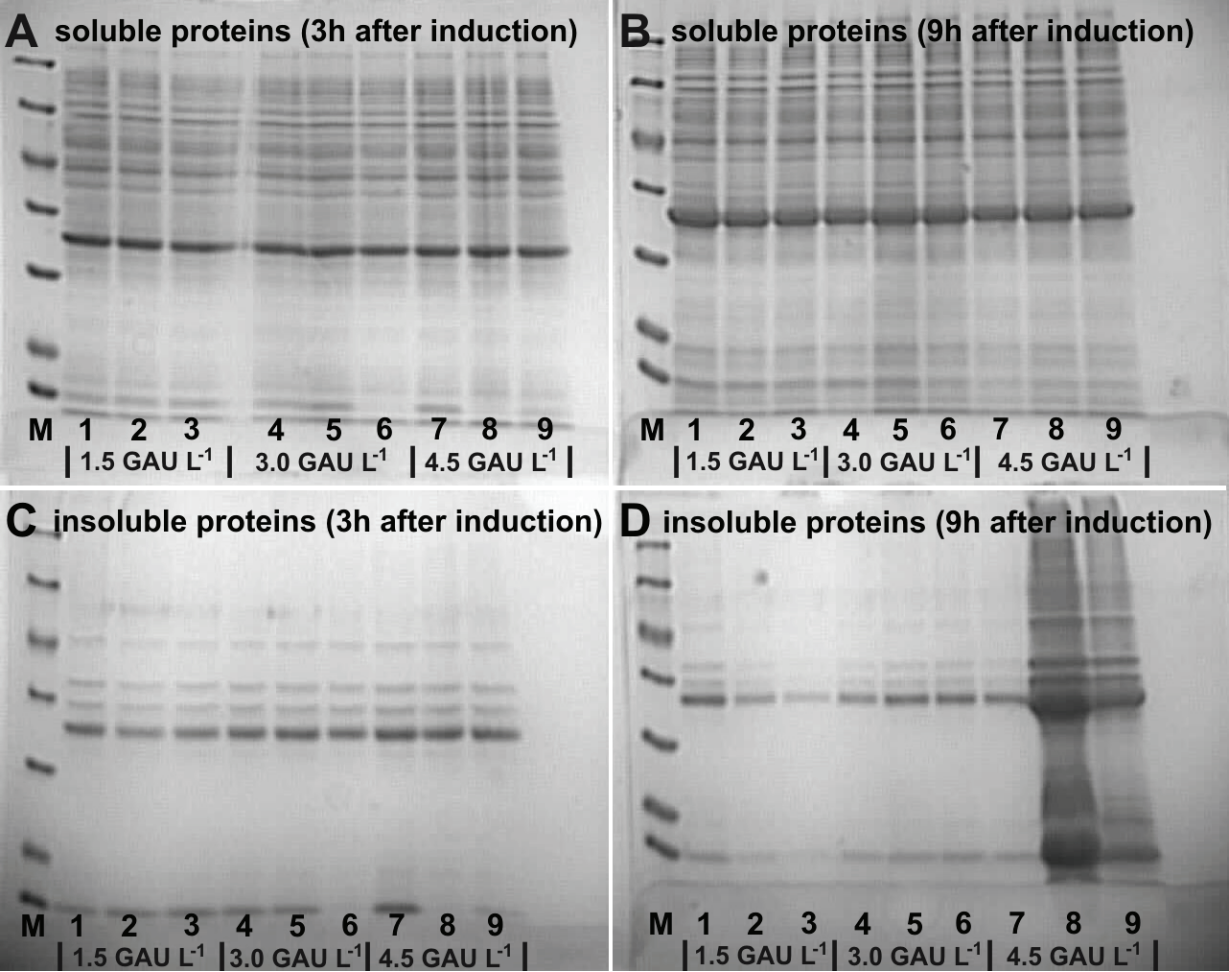
**The comparison of heterologous alcohol dehydrogenase expression in *E. coli *RB791 *pAdh *cells with supplementation of PFD at the time of induction**. Data are shown for the cultures which were supplemented with PFD saturated by air or PFD saturated by air enriched by 40% v/v of O_2_. M - molecular weight marker; lanes 1, 4, 7 - cells cultured without PFD (PFD_); lanes 2, 5, 8 - cells cultured with PFD saturated by air (PFD_+_); lanes 3, 6, 9 - cells cultured with PFD saturated by air and enriched by 40% v/v of O_2 _(PFD_40%O2_); concentrations of enzyme are shown under the numbers of the analyzed samples.

After addition of the PFD phase, booster mix and additional enzyme we observed a clear effect of PFD on the further growth, dependent on the added enzyme concentration. Without PFD in all instances the final OD_600 _was significantly lower compared to cultures with PFD. The highest cell concentrations in the PFC containing cultures with a final concentration of glucoamylase in the range of 1.5 to 4.5 U L^-1 ^were about 40% higher compared to the cultures which were not supplied with PFD. The highest cell density was obtained in the presence of PFD enriched with 40% v/v of pure oxygen at a concentration of 4.5 U L^-1 ^of glucoamylase. The OD_600 _values of the cultures supplemented with PFC saturated with pure oxygen were higher than those measured for control cultures, but significantly lower than the densities which were obtained with a perfluorinated phase which was enriched with 40% v/v of pure O_2 _(cf. Figure 2). All cultures produced the model protein ADH in high concentration. The amount of soluble product per cell was the same in all cultures, independent on the amount of PFD (shown exemplary for the cultures with 1.5, 3.0, and 4.5 units of amylase for PFD saturated with air or 60% v/v of oxygen in Figure 3) and thus, the final volumetric product yield was highest in the cultures which also showed the highest cell density according to Figure 3.

## Discussion

PFC supplementation of microbial cultures has been in the past applied to improve the oxygen transfer [2-4]. However, the application of PFCs was mainly limited to its relatively high price and the relatively small effect on fermentations, where other alternatives for increasing the oxygen transfer exist.

Recently there are many activities to apply fed-batch technologies under miniaturised culture conditions [17-20], as process development can be significantly shortened by applying already at this scale conditions which are typical for the industrial processes [21]. In contrast to the bioreactor scale, where *k*_*L*_*a *values are relatively high and additionally oxygen can be supplied to the gas phase, oxygen limitation is a major bottleneck in micro-scale cultures. Especially microwell and deepwell plates have lower *k*_*L*_*a *values [22,23], and under these conditions also the price for the application of PFCs is not any more a limitation due to the small volumes which are needed. Thus it was a major aim in the actual study to investigate which benefit is possible by the use of PFC's in ml-scale cultures.

Our results show that in deepwell plate cultures PFD can provide a severe growth benefit and even can improve the yield of correctly folded product. For this however, PFCs should be not applied at the early culture phase, as under these conditions the oxygen would be delivered already during a time where oxygen diffusion from air is not limiting the culture growth. Over the long time of cultivation the excess oxygen from the carrier can diffuse into the culture medium and finally to the air phase and thus the oxygen-enriched PFD is not effective. But, we could show that PFD addition is very beneficial after a high cell density has been reached at the time of induction, when it is also known that the cells have a high respiration requirement [24]. Under these conditions the biomass yield could be increased by 40% and thus the volumetric yield of soluble ADH also increased (cf. Figure 3).

A major point which was newly addressed in this paper compared to other fermentation studies where this had been overlooked was the concentration of oxygen in the PFD phase. Too high oxygen concentration resulted in growth inhibition. In the tests performed here an optimum was found where the PFD is enriched by 40% v/v of pure O_2_. High pO_2 _levels in the culture medium could provoke the increase of H_2_O_2 _or oxygen radicals, and consequently may lead to oxidative stress and membrane damage [25,26]. The inhibitory effect of concentrated oxygen has been observed previously for plant cells cultured in the presence of oxygen loaded PFD [10].

It is generally considered in batch cultures, that the cell density of induction in shaken cultures is a very critical parameter for protein production. Slight changes in the time of induction, i.e. cell density, have big effects. Thus it was surprising, that in the case of added PFD, good accumulation of ADH was obtained in all cultures, i.e. in this case the time of induction was not any more a critical parameter and thus cultures were much more robust. This is similar to fermentation processes, where with a good supply of medium components and the control of the culture growth rate by the carbon source, i.e. application of the substrate limited fed-batch strategy, the process is very robust in connection to the induction cell density (see e.g. [21]).

High expression of heterologous ADH encoded by the *pAdh *plasmid has been observed in all cultures supplemented with or without PFD-based oxygen carrier. However, it is remarkable that the amount of ADH in the insoluble protein fraction was lower in the cultures with 1.5 GAU L^-1 ^additionally added enzyme with high amount of loaded oxygen to PFD. This may be due to the higher turnover of inclusion bodies as earlier described e.g. for α-glucosidase [27], which is dependent on ATP. This observation is interesting and will be further investigated.

## Conclusions

The results of our experiments have confirmed the feasibility and benefit of perfluorinated oxygen carrier application in high cell density cultures of *E. coli *at the example of miniaturised cultivation systems. Supplementation of the miniaturized fed-batch system (as EnBase-Flo) with aerated/oxygenated PFD had positive effects on *E. coli *RB791 *pAdh *cells cultivated to reasonable high cell density. The oxygen containing PFC phase enhanced the growth of *E. coli *cells and higher OD_600 _values were measured in cultures supplemented with this oxygenated liquid oxygen carrier compared to control cultures without any PFD added. The highest value of cell density has been noted when cultures were supplemented with PFD saturated by air and mixed in a 60/40% v/v ratio with O_2 _saturated PFD. Interestingly the improved oxygen supply did not only increase the cell density, but also resulted in a higher volumetric yield of the target protein.

Our results indicate that PFD-based oxygenation systems may be valuable additives in miniaturized cell culture formats. The positive effect of PFD is even more obvious when is added during high cell density cultures and the effect depends on the amount of oxygen loaded to the PFD.

## Methods

### Bacterial strain

The recombinant strain of *Escherischia coli *RB791 *pAdh *[28] encoding a heterologous alcohol dehydrogenase was used in this work. The gene of an alcohol dehydrogenase (*adh*) from *Lactobacillus spec*. was cloned into the pQE30 plasmid (Qiagen, USA) yielding the vector pQE30*adh *and the protein was expressed in *E. coli *RB791 [F^-^, IN(*rrnD*-*rrnE1*), λ^-^, *lacI*^*q*^*L*_*8*_] kindly provided by the *E. coli *Genetic Stock Center (New Haven, USA).

### Cultivation media

Two different media were used for the cultivations: (1) EnBase-Flo Mineral Salt Medium (MSM) and (2) EnBase-Flo Complex Medium (CM). Both of them were obtained from BioSilta Oy (Oulu, Finland) and have been earlier introduced by Krause et al. [13]. The MSM and CM had the following composition: Na_2_SO_4 _(2.0 g L^-1^), (NH_4_)_2_SO_4 _(6.12 g L^-1^), NH_4_Cl (0.50 g L^-1^), K_2_HPO_4 _(14.60 g L^-1^), NaH_2_PO_4_·H_2_O (3.60 g L^-1^), (NH_4_)_2_-H-citrate (1.00 g L^-1^), MgSO_4 _(3 mM), thiamine hydrochloride (0.1 g L^-1^) and 2 ml trace element solution (containing per litre: 0.50 g CaCl_2_·2H_2_O, 0.18 g ZnSO_4_·7H_2_O, 0.10 g MnSO_4_·H_2_O, 20.1 g Na_2_-EDTA, 16.70 g FeCl_3_·6H_2_O, 0.16 g CuSO_4_·5H_2_O and 0.18 g CoCl_2_·6H_2_O). Both used media differed in level of organic nitrogen compounds and were further supplemented with complex medium additives (optimized combination of peptones and yeast extracts) commercially named as EnBase Boosting mix (BioSilta Oy): CM had a 10 times higher concentration of complex additives then MSM.

The LB medium (10 g L^-1 ^Trypton, 5 g L^-1 ^yeast extract and 5 g L^-1 ^NaCl; pH was set to 7.0) was used for inoculum preparation.

### Perfluorinated oxygen carrier

Perfluorodecalin (PFD; C_10_F_18_; 1,1,2,2,3,3,4,4a,5,5,6,6,7,7,8,8,8a-octadecafluorodecalin, ABCR GmbH, Karlsruhe, Germany) was used as a liquid oxygen carrier in this work. According to the information of the supplier the applied PFD is a 98% m/m equimolar mixture of (cis-/trans-) isomers. The density of used liquid PFD is 1.941 kg L^-1 ^(at 25°C), dynamic viscosity is 5.1 mPa · sec (at 25°C) and surface tension is 1.6 Pa. The used PFD did not disperse and remained at the bottom of wells during all experiments.

PFD was sterilized by autoclaving, cooled to 37°C and then PFD was saturated by compressed air and pure oxygen in aseptic conditions supplied with a 0.2 μm cartridge filter to prevent microbial contamination [10]. It is known that oxygen solubility in PFD does not vary significantly with temperature [29]. 4.0 mM O_2 _(0.128 g O_2_) from air [30] or 19.2 mM O_2 _(0.614 g O_2_) from pure oxygen can be dissolved in 1 L of PFD at 37°C [29]. Different kinds of PFD mixtures were used during the experiments and the calculated values of oxygen concentration in used PFD mixtures are presented in Table 1.

**Table 1:** Mixtures of PFD used during experiments and total content of oxygen in the liquid oxygen carrier

Symbol	Composition of PFD mixture (per 1.0 ml)	**Content of oxygen [μM O**_**2 **_**(ml**^**-1 **^**PFD)]**		
		
		from air	from pure oxygen	total
**PFD**_**air**_	1.0 ml PFD_air_	4.00	0	**4.00**

**PFD**_**20%O2**_	0.8 ml PFD_air _+ 0.2 ml PFD_O2_	3.20	3.84	**7.04**
**PFD**_**40%O2**_	0.6 ml PFD_air _+ 0.4 ml PFD_O2_	2.40	7.68	**10.08**
**PFD**_**60%O2**_	0.4 ml PFD_air _+ 0.6 ml PFD_O2_	1.60	11.52	**13.12**
**PFD**_**80%O2**_	0.2 ml PFD_air _+ 0.8 ml PFD_O2_	0.80	15.36	**16.16**
**PFD**_**O2**_	1.0 ml PFD_O2_	0	19.20	**19.20**

The oxygen concentration in PFD was calculated based on data published by Costa Gomez et al. [29] and Amaral et al. [31].

Unsaturated perfluorodecalin (PFD_+_) was also used in reference experiments to evaluate possible side effects triggered by the perfluorochemical. The culture which was not supplemented with PFD (marked as PFD_-_) was also used as reference. PFD created a separate phase on the bottom of the wells after pipetting as it cannot mix with aqueous systems. Thus, the volume of PFD which was added to the culture medium does not change the concentration of its ingredients.

### Cultivation

The *E. coli *RB791 *pAdh *inoculum was prepared from a overnight Petri dish culture with LB agar at 30°C. Precultures were washed from the plate with 3.0 ml of fresh MSM and after OD_600 _measurement the suspension of *E. coli *cells was used as inoculum. All cultures were started with an OD_600 _of 0.15.

Cultivations of *E. coli *RB791 *pAdh *were performed in square-bottom 24-deep-well plates (DWP). The initially culture volume was 2.5 ml. The DWPs were covered with a gas-permeable membrane (Easy-Breath Self Adhesive Sheets; BioSilta Oy) to provide aeration during cultivation. All cultures were incubated at 30°C on orbital shakers (250 rpm, 2.5 orbit, Kuhner) and cells were precultivated for 2 hours before the initial addition of 1.5 U L^-1 ^of glucoamylase (GA). Enzyme was not added immediately after inoculation in order to prevent the immediate release of glucose subunits from the carbohydrate polymer.

After 15 hours of cultivation the cultures were induced with 1 mM of isopropyl β-D-1-thiogalactopyranoside (IPTG; Carl Roth GmbH, Karlsruhe, Germany). At the same time the cultures were supplemented with 0.6 ml of EnBase Boosting Mix as recommended by the supplier (BioSilta), and simultaneously also 3.0 ml gas-saturated PFD was added to the cultures and additional amounts of GA as indicted in the Results section to obtain final concentrations of the amylolytic enzyme of 1.5, 3.0, 4.5, and 6.0 U L^-1^.

100 μl samples were harvested to measure the OD_600 _and eventually for SDS-PAGE analysis of soluble and insoluble proteins. The average rate of water evaporation from the cultures through the air permeable membrane was calculated as 9.1 μl h^-1 ^for every used well of DWP. All experiments were performed twice and the shown OD_600 _values are an average of the measured values of the single cultures.

### Analytical methods

Cell growth was followed with a spectrophotometer after the dilution of harvested culture samples in culture medium at 600 nm (OD_600_). All cell density values are presented as OD_600 _values. For non-induced cells the OD_600 _value of 1 is equivalent to 1.7 g L^-1 ^of wet cell weight and to 0.39 g L^-1 ^of dry cell weight of *E. coli *RB791 *pAdh *biomass. This relation slightly changes after induction.

### Protein analysis

Samples for protein analysis were harvested at 9 h after IPTG addition. All samples were standardized to equal OD_600 _values of 10. Therefore, protein productivity per cell in different samples can be directly compared on the basis of the protein band thickness. Standardized samples were pelleted by centrifugation (13,000 × g, 5 min, 4°C), supernatants were discarded and the pellets were frozen at -20°C. For analysis, each cell pellet was resuspended in 1.0 ml 0.1 M Tris/HCl in 1.0 mM EDTA buffer (pH 7.0) and 2.0 μl of lysozyme (50 mg mL^-1^) was added. After incubation on ice for 30 min, the resuspended pellets were disrupted by sonication on ice (UPS200, sonotrode with 2 mm diameter, 70% power input, Dr. Hielscher GmbH, Germany) in 3 × 30 sec periods with 45 sec cooling breaks in between. To obtain the soluble and insoluble protein fractions, the disrupted cell solution was centrifuged (13,000 × g, 5 min, 4°C) and an aliquot for the soluble protein analysis was drawn from the supernatant. The pellet was also harvested for analysis of the insoluble protein fraction. The supernatant was directly used as soluble protein sample. The pellets of the insoluble protein fraction were suspended in 1.0 ml of 0.1 M Tris/HCl in 1.0 mM EDTA buffer (pH 7.0) before analysis. The soluble and insoluble fractions were analyzed by SDS-PAGE on 12% m/m polyacrylamide gels (60 V for 30 min followed by 90 V for 90 min). The bands were visualized by Comassie Brillant Blue R 250 solution according to standard staining method modified by Besir's [30].

## Competing interests

MP and JG declare that they have no competing interests. PN is an adviser to BioSilta Oy.

## Authors' contributions

MP carried out most of the experiments, did the analyses and drafted the manuscript. JG participated in the experiments with the DWPs. PN supervised the study, and participated in its design and coordination, helped to draft and writing of the manuscript. All authors read and approved the final manuscript.
